# Fruit resources shape sexual selection processes in a lek mating system

**DOI:** 10.1098/rsbl.2024.0284

**Published:** 2024-09-25

**Authors:** H. Luke Anderson, Jairo Cabo, Jordan Karubian

**Affiliations:** ^1^ Department of Ecology and Evolutionary Biology, Tulane University, New Orleans, LA 70118, USA; ^2^ Fundación para la Conservación de los Andes Tropicales, Quito, Ecuador; ^3^ Universidad San Francisco de Quito USFQ, Estación de Biodiversidad Tiputini, Quito, Ecuador

**Keywords:** sexual selection, resource availability, display rate, manakins, hotspot hypothesis, lek paradox

## Abstract

The degree to which within-population variation in sexual trait expression relates to resource heterogeneity remains poorly explored. This is particularly true in lek-mating species, where genetic explanations for male phenotypic variance and mating success are dominant. Here, we demonstrate a link between fine-scale fruit resource availability and indices of male mating success in the white-bearded manakin (*Manacus manacus*), a lek-mating frugivorous bird that produces energetically costly courtship displays. We used motion-activated camera traps to monitor male display behaviour and female visitation at male courts while concurrently conducting twice-monthly fruit surveys around courts. We observed significant variability in ripe fruit biomass among display courts and leks, and mean fruit biomass at courts significantly predicted male display rates. In turn, male display rate was the strongest predictor of female visitation to courts. Causal modelling supported the hypothesis that hyper-local fruit availability indirectly affects female visitation via its direct effects on male display rate. The demonstration that resource availability at fine spatial scales predicts display rate in a lekking organism—for which resource-related variables are typically not considered to play important roles in shaping male reproductive variance—has implications for the expression, honesty and maintenance of sexually selected traits under fluctuating ecological conditions.

## 1. Introduction

Despite recent calls to better integrate the field of sexual selection with ecology [1–5], connections between environmental factors and sexual selection processes remain understudied, and gene-centric explanations for variation in male sexual trait expression and mating success remain the default [1,6]. The relative neglect of environmental factors in sexual selection research may represent an important knowledge gap [1], as resource availability is known to shape individual condition [7,8] and, in turn, male investment in costly ornaments or displays [8–11]. More broadly, the degree to which environmental heterogeneity shapes trait expression within populations has implications for male signal honesty [12], the adaptive value of female preferences [13] and the maintenance of genetic variation in sexual traits [14]. Thus, resolving linkages between environmental factors and sexual selection processes may advance our understanding of the evolution and maintenance of sexual traits in nature.

The emphasis on genetic over environmental factors has been particularly pronounced in studies of lek-mating organisms, where males aggregate to perform courtship displays [15] and often exhibit high variance in mating success [16–19]. Because males in these systems provide no direct benefits to females beyond their sperm [15], female choice is thought to be driven primarily by indirect genetic benefits (i.e. genes that increase offspring survival and/or attractiveness [8,20,21]). Nevertheless, there are several reasons to expect male variance in display behaviour and mating success on leks to be linked to environmental factors like resource availability. One of the early hypotheses for the evolution of leks—the ‘hotspot hypothesis’—supposes that leks form in locations where males are more likely to encounter females, such as areas of high resource density [22,23]. By extension, if resources vary among male territories within a lek or population, males in relatively resource-rich territories may be expected to experience higher rates of female visitation. In addition to potential benefits related to female space use *per se*, males with display sites situated near abundant food resources may be able to allocate more energy to costly display behaviour [24–26] or forage more efficiently due to greater resource proximity, allowing them to spend more time on the lek [27]. To date, however, the degree of variation in resource availability within populations and its relation to individual-level display behaviour and mating success has rarely been investigated in lekking systems (but see [28,29]).

Here, we investigate relationships between fine-scale fruit availability, male display rate and female preference in a lek-mating frugivorous bird, the white-bearded manakin (*Manacus manacus*). We hypothesized that resource heterogeneity could, in part, explain the high variance in male display rate [25] and mating success [16] observed in *Manacus* manakins and other lekking organisms. Therefore, we set out to test the following associated predictions: first, that male display courts within a population would vary in their surrounding fruit resource availability; second, that courts with greater ripe fruit biomass in their immediate vicinity would exhibit higher rates of male display, as court owners would be able to allocate more time and energy to display behaviour due to the combined effects of resource proximity and abundance; and third, that courts with higher fruit abundance and/or male display rates would exhibit higher rates of female visitation (a proxy for mating success [28,30,31]). To address these predictions in our study population of *M. manacus*, we surveyed fruit biomass around male display courts while concurrently monitoring rates of male display and female visitation during a three-month period of peak breeding activity in northwest Ecuador.

## 2. Methods

### (a) Study system

The white-bearded manakin (*M. manacus*) is a lek-mating frugivorous bird found in South American secondary and gallery forests. *Manacus* leks are composed of spatially clustered display courts, which males create by clearing debris from small patches of the forest floor [32–34]. At courts, males perform explosive and energetically costly displays, during which heart rates reach among the highest known in vertebrates [25], and court-holding males exclude other adult males from the areas immediately surrounding their courts [35,36]. Male reproductive skew in *M. manacus* is among the most extreme reported in lekking birds [16], and male mating success has been found to correlate with body size, condition, court centrality and male–male aggressive behaviour [37]. In the sister taxon *M. vitellinus*, both the overall display rate and the performance of precise display components have been implicated as targets of female preference [38]. Male *Manacus* manakins have been reported to spend as much as 90% of their time at courts during breeding periods [23,26,34], suggesting that resources immediately surrounding courts may be important in shaping male display behaviour.

We studied five leks at Reserva FCAT (Fundación para la Conservación de los Andes Tropicales), a private reserve in Esmeraldas Province, Ecuador. In our population (subspecies: *M. m. leucochlamys*), focal leks included between three and 10 courts, and nearest-neighbour court distances within leks ranged from 2 to 102 m (mean ± s.e. = 29.0 ± 5.1 m). We considered males displaying within visual or auditory distance of one another as belonging to the same lek (per [28,39,40]).

Data collection occurred between 19 November 2021 and 15 February 2022, during peak display season at our site. Cameras were placed at all known courts at the start of the data collection period and added to additional courts as they were discovered. After excluding courts that were established late in the study, inactive or used very sporadically (electronic supplementary material, figure S1), the remaining courts (*n* = 25) were used in analyses of fruit availability. We obtained a median of six fortnightly fruit surveys at focal courts during the study period (mean ± s.e. = 5.36 ± 0.23 surveys; range = 3−7). Five additional courts were excluded from behavioural analyses due to insufficient camera monitoring or improper camera placement. Thus, the final behavioural dataset included 20 courts across five leks, which were monitored with camera traps for a median of 69 days (mean ± s.e. = 66.3 ± 2.0 days; range: 47−85).

### (b) Behavioural monitoring

We used Browning Recon Force Elite HP4 motion-activated trail cameras to monitor behaviour at male display courts, where key female-directed courtship activities in *M. manacus* are known to occur [16,35]. Cameras were placed 1−2 m from courts and checked regularly to confirm sensor functionality. Video length was initially configured to 60 s but later reduced after two weeks of monitoring revealed that activity bouts did not typically extend beyond 30 s. The refractory interval between videos was 1 s and monitoring occurred for all daylight hours.

Videos were manually reviewed to count instances of male display (i.e. jump-snaps, jump-snips, grunt-jumps, roll-snaps; described in [34,41–43]) and female visitation. Male display bouts vary in length, so we counted the total number of display components produced within display bouts rather than the number of bouts. The overall display rate for a given court was calculated by summing display component counts and dividing by the number of days a court was monitored. Because first-year male and adult female bearded manakins have similar dull-green plumage, we used a combination of behavioural cues and colour band identities (IDs) to distinguish between instances of female or juvenile male presence in videos. Instances where green-plumaged birds could not be confidently identified as females or juvenile males were excluded from analyses, and juvenile male practice displays were excluded from display rate calculations.

### (c) Fruit surveys

We conducted twice-monthly visual surveys to quantify fruit resources surrounding male display courts. Circular survey plots were centred around male courts with a radius of 10 m (i.e. 314 m^2^ total area). This size was chosen because it encompassed the largest reported territory size for *M. manacus* (202 m^2^) [36] while minimizing the overlap between adjacent plots. Studies of *M. manacus* indicate that they are highly generalist frugivores, consuming the vast majority of available fleshy fruit species [34,44,45] smaller than 17 mm in diameter [44]. Thus, during surveys, we recorded all ripe fleshy fruits below this size. Visual surveying of all forest strata was possible because leks occur in regenerating secondary forest habitats with low canopy heights (approx. 10−15 m maximum), and nearly all relevant fruits detected in survey plots were borne by understory shrubs, treelets and lianas. Most belonged to the families Melastomataceae and Rubiaceae (76% and 16% of total biomass, respectively), which are the dominant plants in white-bearded and other manakin diets [34,46] and similar in macronutrient profile and caloric content [47,48]. Fruits were identified to morphospecies prior to the start of the study, and a representative sample of each morphospecies (approx. 10−20 berries) was collected and weighed to calculate an average wet weight per fruit [17,28]. To estimate fruit biomass for a given plant, we counted the number of ripe fruits and multiplied it by the average weight for the morphospecies [49]. The total ripe fruit biomass across all plants in a survey plot was then summed to produce our estimate of fruit availability surrounding a court at a given time point.

### (d) Spatial and habitat characteristics

In addition to surveying fruit resources, we also collected data on non-resource-related spatial and environmental variables hypothesized to relate to rates of male display or female visitation at courts. These variables included canopy cover, which may influence the ambient light environment important for male signalling [50–52] or susceptibility to aerial predators [53] and thus time dedicated to vigilance behaviour [54,55]; lek centrality, which has been implicated in female choice and male–male competition in numerous lekking systems, including white-bearded manakins [27,37,55–57]; and nearest-neighbour distance, which could influence male display rate through social stimulation or competition [58,59] or correlate with visitation rate due to female preferences for clustered males [31,40,60,61]. Centrality and nearest-neighbour distances may also be influenced by fruit availability and canopy cover if males cluster in or around desirable sites.

To assess spatial variables, we obtained UTM coordinates for each display court using a handheld GPS. We then imported points into QGIS v. 3.16.4 [62] to obtain straight-line distances to nearest neighbours and the lek centroid. In nearest-neighbour calculations, only active courts were considered; for centrality calculations, the lek centroid was defined as the unweighted centre of the minimum convex polygon of the constituent display courts. To obtain measures of canopy cover as a proxy for court-level light environment, we took standardized, upward-facing photographs from the centre of the cleared area at male courts at approximately the height at which displays occur (15 cm) and imported images into *Canopeo*, a mobile application for rapid assessment of fractional green canopy cover [63].

### (e) Statistical analyses

Statistical analyses were conducted in R v. 4.3.3 [64]. To assess whether fruit biomass varied across male courts and leks, we fit linear mixed-effects models (LMMs) using the package *nlme* [65]. The court-level model included fruit biomass at each display court as a response variable, display court ID as a predictor variable, and lek ID and survey date as random intercepts. The lek-level model included fruit biomass at each display court at a given time point as a response variable, lek ID as a predictor variable, and display court ID and survey date as random intercepts. Fruit biomass was transformed using log(*x* + 1) to improve residual normality.

To assess whether resource availability predicted male display and female visitation rates, we fit LMMs in *nlme* that included lek ID as a random intercept and a spatial autocorrelation structure (corSpher with a nugget effect). We explicitly accounted for spatial autocorrelation because some survey plots in the largest lek overlapped [66,67]. Variables were centred and scaled before analysis, and we employed data transformations to reduce outlier influence and improve residual normality; untransformed data yielded qualitatively similar results. The display rate model included natural log-transformed male display rate as a response variable and mean fruit biomass as a predictor variable; the female visitation model included natural log-transformed female visitation rate as the response variable and mean fruit biomass and natural log-transformed male display rate as predictors. We used an overall measure of male display rate in analyses (i.e. the sum of all jump-snaps, jump-snips, grunt-jumps and roll-snaps produced on the court per day of monitoring) because all display components are used in female-directed courtship [68], impose similar energetic costs [25] and were hypothesized to relate to resource availability. The overall display rate was tightly correlated with the jump-snap rate (*r* > 0.99)—the most common display component produced on courts—and qualitatively similar results were obtained when jump-snaps alone were analysed.

Finally, we used causal modelling to further evaluate relationships between exogenous variables of interest (fruit biomass and canopy cover) and endogenous variables (fruit biomass, centrality, nearest neighbour distance, male display rate and female visitation rate). In causal modelling, endogenous variables are those that are hypothesized to be caused by other variables within the model; exogenous variables have causes outside the model [69]. We fit path models using the *lavaan* package in R [70], and the full model comprised four regressions: male display rate as a function of mean fruit biomass, canopy cover, centrality and nearest-neighbour distance; female visitation rate as a function of male display rate and all spatial and environmental variables and centrality and nearest-neighbour distance each as a function of mean fruit biomass and canopy cover. Variables were centred, scaled and transformed (natural log transformation of male display rate and female visitation rate, square root transformation of nearest-neighbour distance) to meet assumptions of underlying regression models and reduce outlier influence. The full model was winnowed by removing single variables (without suppressing conceptually important paths) and assessing whether the model fit improved according to a variety of commonly used indices [29,71], including *Χ* ^2^, root mean square error of approximation (RMSEA), standardized root mean square residual (SRMR), comparative fit index (CFI) and Tucker–Lewis index (TLI) [72–75].

## 3. Results

### (a) Heterogeneity in fruit availability across courts and leks

**Figure 1.**
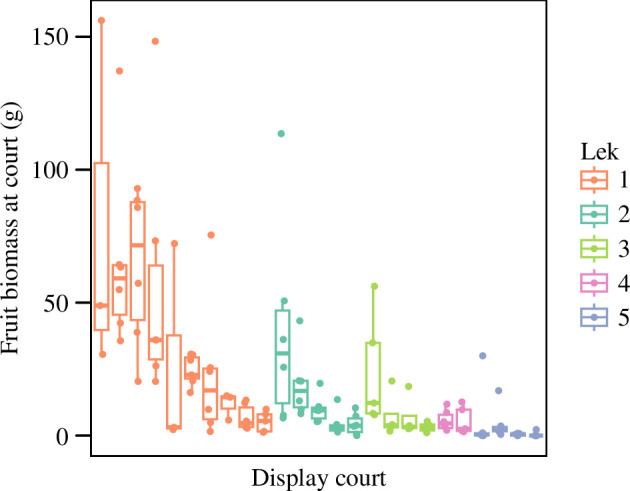
Fruit biomass varied significantly between male display courts (LMM: *F* = 6.77, d.f. = 24, *p* < 0.0001) and leks (LMM: *F* = 6.54, d.f. = 4, *p* = 0.002). Points represent ripe fruit biomass within a 10 m radius of monitored display courts based on fortnightly surveys throughout the study period.

We observed considerable variation in fruit availability across male display courts, with some harbouring significantly higher mean fruit biomass in their vicinity than others (LMM: *F* = 6.77, d.f.= 24, *p* < 0.0001; figure 1). On average, the most resource-rich court contained >200 × more fruit in the surrounding area than the least resource-rich court. There was also significant variation in fruit biomass per court between different leks (LMM: *F* = 6.54, d.f. = 4, *p* = 0.002; figure 1).

### (b) Fruit biomass predicts male display rate at courts

**Figure 2.**
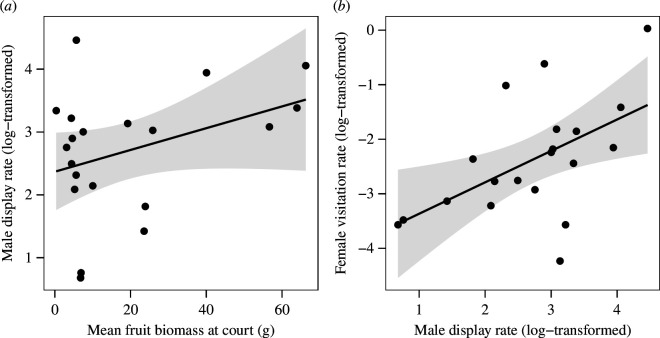
(*a*) Mean fruit biomass significantly predicted male display rate (displays per day) at courts (LMM: *t* = 3.01, d.f. = 14, *p* = 0.009). (*b*) Male display rate significantly predicted female visitation rate (visits per day) to courts (LMM: *t* = 3.67, d.f. = 14, *p* = 0.003). Shaded areas represent 95% confidence intervals.

Supporting the hypothesis that local resource availability positively influences male energy or time budgets for display, mean fruit biomass significantly predicted male display rate at courts (LMM: *t* = 3.01, d.f. = 14, *p* = 0.009; *R* ^2^_marginal_ = 0.22, *R* ^2^_conditional_ = 0.47; figure 2*a*).

### (c) Male display rate is the strongest predictor of female visitation at courts

In a multiple regression model including both male display rate and mean fruit biomass as predictors of female visitation to courts (LMM: *R* ^2^_marginal_ = 0.18, *R* ^2^_conditional_ = 0.74; ∆AICc = 4.86), male display rate more strongly predicted visitation (*t* = 1.96, d.f. = 13, *p* = 0.07) than mean fruit biomass (*t* = 1.51, d.f. = 13, *p* = 0.15). When male display rate alone was included as a predictor, which was the model favoured by ∆AICc, male display rate significantly predicted female visitation (*t* = 3.67, d.f. = 14, *p* = 0.003; *R* ^2^_marginal_ = 0.24, *R* ^2^_conditional_ = 0.67; ∆AICc = 0; figure 2*b*). However, a less well-supported model with mean fruit biomass alone as a predictor (*R* ^2^_marginal_ = 0.12, *R* ^2^_conditional_ = 0.76; ∆AICc = 1.84) also significantly predicted visitation (*t* = 3.87, d.f. = 14, *p* = 0.002), justifying further exploration of these relationships using path analysis (below).

### (d) Path analysis

**Figure 3.**
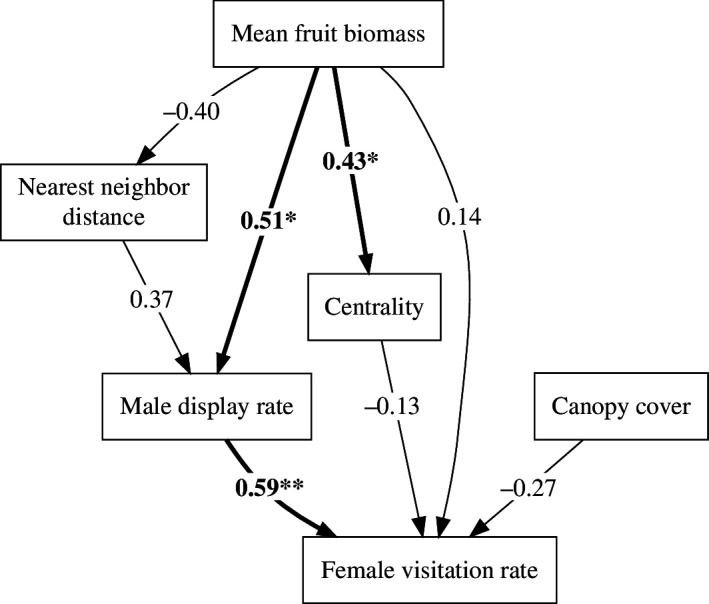
Path analysis supported the hypothesis that fruit biomass directly influences male display rate, which in turn directly influences visitation rate. Edges are labelled with standardized path coefficients. Bold lines and asterisks denote significant paths (* = *p* < 0.05; ** = *p* < 0.01).

The final path model (figure 3; electronic supplementary material, figure S2) exhibited a good fit based on a variety of indices (RMSEA = 0.05, CFI = 0.98, TLI = 0.95, *Χ* ^2^ = 0.39, SRMR = 0.09). Male display rate at courts was significantly predicted by mean fruit biomass and non-significantly predicted by nearest-neighbour distance (table 1). Female visitation rate was significantly predicted by male display rate and non-significantly predicted by canopy cover, centrality and mean fruit biomass. In addition, mean fruit biomass at courts was a significant predictor of centrality and a marginally significant predictor of nearest-neighbour distance. Direct effects of fruit biomass on female visitation were estimated to be weaker than indirect effects mediated by male display rate (figure 3; table 2).

**Table 1.** Direct effects in the final path model.

response variable	predictor variable	std path coefficient	*z*	*p*	*R* ^2^
log(male display rate)	mean fruit biomass	0.51	2.40	0.02 *	0.42
	sqrt(nearest neighbour distance)	0.37	1.73	0.08	
log(female visitation rate)	log(male display rate)	0.59	3.23	0.001 **	0.24
	canopy cover	−0.27	−1.49	0.14	
	centrality	−0.13	−0.70	0.48	
	mean fruit biomass	0.14	0.67	0.50	
centrality	mean fruit biomass	0.43	2.11	0.04 *	0.18
sqrt(nearest neighbour distance)	mean fruit biomass	−0.40	−1.94	0.05	0.16

**Table 2.** Direct versus indirect effects of fruit biomass on female visitation.

effect type	estimate	s.e.	*z*	*p*
indirect	0.31	0.16	1.93	0.05
direct	0.15	0.22	0.67	0.50
total	0.46	0.24	1.86	0.06

## 4. Discussion

We show that the availability of fruit—long hailed as an easily obtained and non-limiting resource akin to a ‘free lunch’ [76–78]—varies dramatically across fine spatial scales and predicts rates of male display in a lek-mating bird. In turn, rates of male display (but not fruit availability *per se*) predict female visitation, which has been shown to correlate strongly with ultimate male mating success in bearded manakins [30] and other lekking species [31]. These findings extend our understanding of lek mating systems and sexual selection in several important ways. First, we demonstrate that biologically relevant resource hotspots can occur at smaller spatial scales than previously recognized. Second, rather than directly increasing female encounter rates as proposed by the classic hotspot hypothesis [22,23,79], we show that males may benefit from proximity to fruit resources due to its effects on their own display behaviour. Third, these findings—particularly in the context of a classical lek mating system, which are often considered paragons of indirect benefits mate choice models—suggest that within-population resource heterogeneity may play an underappreciated role in shaping male display rate variance and sexual selection processes more broadly.

We propose two major interpretations for the correlation between fruit biomass and male display rate in our system. One possibility is that male settlement is largely stochastic, and court-level resource availability generates the observed variance in male display rate by enabling males at high-fruit courts to maintain better body condition, allocate more energy to display and/or spend less time foraging due to greater resource abundance and proximity. A second possibility is that higher quality males (i.e. those with greater display capacity) outcompete lower quality males for access to resource-rich courts [28], and thus intrinsic differences between males (possibly driven by ‘good genes’) shape the observed patterns. If better males obtain better sites, proximity to fruit may also serve as an honest indicator of male quality [28,80]. In either scenario, results imply that individual males benefit from holding resource-rich display sites, which has not been demonstrated in classical lekking systems. Importantly, these two possibilities are not mutually exclusive and may even form a feedback loop: if high-quality males obtain high-quality sites, greater resource availability at these sites could amplify pre-existing individual differences between males, offering a novel mechanism for generating the extreme variance in male display rate and mating success often observed at leks [25,81–84].

Connections between resource availability and sexual selection processes have implications for the temporal dynamics of male signal honesty and the maintenance of genetic variation under strong selection. For instance, display rate may less reliably indicate male genetic quality during periods of high landscape-level resource abundance (when all individuals can forage with similar efficiency) than during periods of resource scarcity. In systems where key resources are patchy and ephemeral (e.g. rapidly ripening and senescing fruit), we should expect dynamic and complex interactions between male genotypes and environments. Importantly, such interactions may obscure links between male genotypes and sexual phenotypes [14], potentially reducing females’ ability to assess male quality and contributing to the maintenance of genetic variation under strong sexual selection [3,4,14]. These considerations point to the need for longitudinal studies that rigorously track fine-scale ecological variables in relation to male performance and sexual selection processes over longer timescales [2–4], offering a fruitful avenue for future research in sexual selection in this and other systems. Future studies would benefit from directly quantifying foraging by males and females within male territories, monitoring male body condition through time in relation to court-level resource availability, assessing the importance of fruit diversity and nutritional content [85], and experimentally manipulating fruit biomass within male territories.

Finally, we highlight the potential importance of eco-evolutionary feedbacks between frugivory and sexual selection in this system. Frugivorous lekking birds have been shown to disproportionately deposit seeds at leks [86–89], which are highly traditional through time [55,90]; thus, the composition of plant communities at these sites may reflect the consequences of many generations of site-directed seed dispersal. In this study, fruit biomass predicted court centrality and nearest-neighbour distance, suggesting that males may cluster around contemporary resource hotspots, generate resource hotspots over time via seed deposition at display sites, or both. Thus, lekking may constitute a niche construction [91] or habitat-shaping [92] process in frugivorous birds, whereby long-term site-directed seed dispersal modifies the fruit resource environment [93] at leks and incentivizes males to continue settling in traditional areas (e.g. due to foraging and energetic benefits), potentially maintaining male clustering as an evolutionarily stable strategy.

## Data Availability

All data, metadata, and code files used to generate results are archived in the Dryad Digital Repository [94]. Supplementary material is available online [95].
